# QTL/microarray approach using pathway information

**DOI:** 10.1186/1748-7188-7-1

**Published:** 2012-01-15

**Authors:** Hirokazu Matsuda, Yukio Taniguchi, Hiroaki Iwaisaki

**Affiliations:** 1 Graduate School of Agriculture, Kyoto University, Kitashirakawa Oiwake-cho, Sakyo-ku, Kyoto, 606-8502, Japan

## Abstract

**Background:**

A combined quantitative trait loci (QTL) and microarray-based approach is commonly used to find differentially expressed genes which are then identified based on the known function of a gene in the biological process governing the trait of interest. However, a low cutoff value in individual gene analyses may result in many genes with moderate but meaningful changes in expression being missed.

**Results:**

We modified a gene set analysis to identify intersection sets with significantly affected expression for which the changes in the individual gene sets are less significant. The gene expression profiles in liver tissues of four strains of mice from publicly available microarray sources were analyzed to detect trait-associated pathways using information on the QTL regions of blood concentrations of high density lipoproteins (HDL) cholesterol and insulin-like growth factor 1 (IGF-1). Several metabolic pathways related to HDL levels, including lipid metabolism, ABC transporters and cytochrome P450 pathways were detected for HDL QTL regions. Most of the pathways identified for the IGF-1 phenotype were signal transduction pathways associated with biological processes for IGF-1's regulation.

**Conclusion:**

We have developed a method of identifying pathways associated with a quantitative trait using information on QTL. Our approach provides insights into genotype-phenotype relations at the level of biological pathways which may help to elucidate the genetic architecture underlying variation in phenotypic traits.

## Background

In the last decade, a large number of quantitative trait loci (QTL) have been mapped in various organisms, and positional information on QTL is already included in resources such as Online Mendelian Inheritance in Man [1], Mouse Genome Database [2] and AnimalQTLdb [3]. Most previously reported QTL were detected by linkage analyses, but mapping resolutions were too low to identify underlying genes or causative mutations by direct experiments (i.e., the confidence intervals often span tens of map units that contain dozens to hundreds of genes). On the other hand, microarray-based gene expression profiling can be used to discover genes related to a quantitative trait of interest [4]. Combining information on QTL with microarray data enables genes underlying genetically complex traits to be linked to QTL regions. Wayne and McIntyre (2002) [5] proposed the QTL/microarray approach to discover candidate genes. Typically, differentially expressed genes within a QTL region are identified, and candidates revealed based on the known function of a gene in the biological process governing the trait of interest. Because selections are based on limited pre-existing information on what processes may be involved, the nomination of candidate genes is biased [6]. This simplistic approach focuses on gene discovery, which does not provide functional links between biological processes and traits. A low cutoff value in the individual gene analyses (IGAs) may result in many genes with moderate but meaningful changes in expression being missed, leading to a reduction in statistical power [7,8].

Analyzing expression quantitative trait loci (eQTL) in specific tissues is an alternative way to identify potentially causative genes [9,10]. eQTL derived from a polymorphism located near a gene (cis-eQTL) can be distinguished from distant eQTL (trans-eQTL). If cis-eQTL are collocating with phenotypic QTL and their expression is correlated with phenotype, they are regarded as strong candidates [11]. A recent genome-wide association study (GWAS) found that trait-associated SNPs are significantly more likely to be eQTL, and annotating SNPs with eQTL scores can be useful for discovering susceptibility loci [12]. However, the eQTL approach is restricted in its application due to the cost of genome-wide profiling for a large number of animals. In recent years, many methods for transcriptome analyses have shifted the focus from the IGA to a gene set analysis (GSA) to assess the differential expression of pre-defined gene sets that share common biological functions. The GSA has stronger statistical power than the IGA, and been shown to identify many novel gene sets with 'subtle but coordinated' expression patterns [7]. Similar pathway-based approaches have been adopted in GWAS analyses, with some modifications to address the unique challenges of GWAS data [13,14].

The present study aims to identify pathways associated with a quantitative trait of interest using information on QTL. We have developed a pathway-based QTL/microarray approach which provides candidate genes and biological insights into quantitative traits. Our approach is based on a modified GSA, that tests whether a subset of genes within a QTL region is more likely to be relevant to a trait of interest than whole pathway genes. We demonstrate that the combination of gene expression profiling, QTL mapping and GSA produced gene set scores suitable to detect trait-associated pathways.

## Results and discussion

The Mouse Phenome Database (MPD) [15] has developed standards for the deposition of phenotypic data on mice including strain purity, study design, animal age and statistical power. Table 1 shows phenotypic information on male mice for the C3H/HeJ (C3H), C57BL/6J (B6), NZB/BlNJ (NZB) and SM/J (SM) strains from MPD. Measurements are tabulated by strain, sex, strain means, standard deviation and standard error. The plasma high-density lipoprotein (HDL) concentrations of B6 and SM mice were lower than those of C3H and NZB mice. For serum insulin-like growth factor 1 (IGF-1) levels, strains B6 and SM show lower phenotypic values than C3H and NZB mice. Our preliminary study based on the collection of phenotypic data indicated the possibility of identifying genetic differences. For this reason, the present study focuses on comparisons between strains showing high and low phenotypes and identifying significantly (false discovery rate (FDR) < 0.05 and the sub-pathway would be more significant than the whole pathway) altered pathways that are common to the comparisons for each trait.

**Table 1:** Phenotype strain survey data from Mouse Phenome Database

		High-density lipoprotein cholesterol [mg/dL] age 7-9wks				Insulin-like growth factor 1 (adjusted serum IGF-1) [ng/mL] age 15-16wks			
Strain	Sex	Mean	No. of mice	Sd	Se	Mean	No. of mice	Sd	Se
C3H/HeJ (C3H)	Male	111	25	17.7	3.55	123	10	9.77	3.09
C57BL/6J (B6)	Male	72.3	10	9.39	2.97	87.4	10	3.84	1.21
NZB/BlNJ (NZB)	Male	126	10	15.3	4.85	107	10	18.2	5.77
SM/J (SM)	Male	73.2	16	12.2	3.06	60.5	10	23.5	7.44

### HDL cholesterol QTL set

**Figure 1:**
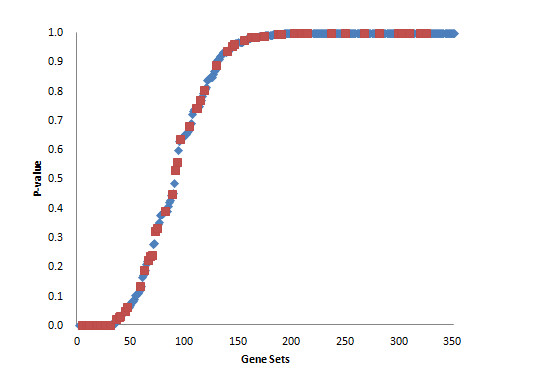
**Sorted *p*-values of gene sets of HDL cholesterol QTL**. Maximum *p*-values in the four comparisons are used. Red (blue) dots indicate the sub- (whole) pathways more significant than the whole (sub-) pathway.

The HDL cholesterol QTL set contained 5,938 genes and 117 pathways. We identified 10 pathways as significantly associated with the trait for four comparisons (Table 2). The sorted maximum *p*-values between four comparisons for HDL gene sets as shown in Figure 1 did not follow the uniform distribution. Although the correlation structure increased the number of false positive, comparing the sub-pathway with whole pathway filter out false positive *p*-values. Out of 10 pathways, 9 were classified in the "Metabolism" category including lipid metabolism (Arachidonic acid and Linoleic acid metabolism) and Drug metabolism - cytochrome P450 pathway in which HDL cholesterol levels increase with increasing liver microsomal P450-enzymes [16,17]. The remaining pathway is the ABC transporter pathway which plays an important role in lipid trafficking. Also, certain genes of ABC transporters have been linked to HDL cholesterol levels. For instance, Abca1 and Abcc6 are annotated with the (mammalian phenotype) MP "decreased circulating HDL cholesterol level" (MP:0000186) in the Mouse Genome Informatics (MGI) database [18]. *Apoa2 *is also known to be involved in the response to HDL cholesterol levels, but its change was relatively small in all comparisons (from 1.00 to 1.04 fold).

**Table 2:** The HDL cholesterol-related pathways detected

	Map category	Direction of change in gene expression*	No. of genes	No. of genes within QTL regions
Drug metabolism - cytochrome P450	Metabolism	B	61	45
Glutathione metabolism	Metabolism	B	48	21
ABC transporters	Environmental Information Processing	D	37	16
Arachidonic acid metabolism	Metabolism	B	63	30
Linoleic acid metabolism	Metabolism	D	33	20
Histidine metabolism	Metabolism	D	22	10
Ascorbate and aldarate metabolism	Metabolism	D	12	11
Pentose phosphate pathway	Metabolism	B	24	10
Propanoate metabolism	Metabolism	D	26	14
Starch and sucrose metabolism	Metabolism	D	26	14

*B and D represent bi-directional and down-regulation, respectively.

### IGF-1 QTL set

**Figure 2:**
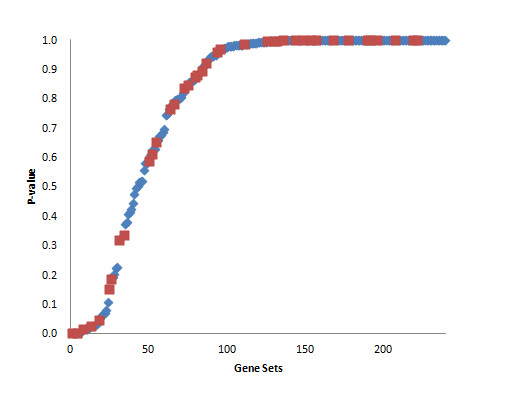
**Sorted *p*-values of gene sets of IGF-1 QTL for four comparisons**. Maximum *p*-values in the four comparisons are used. Red (blue) dots indicate the sub- (whole) pathways more significant than the whole (sub-) pathway.

**Figure 3:**
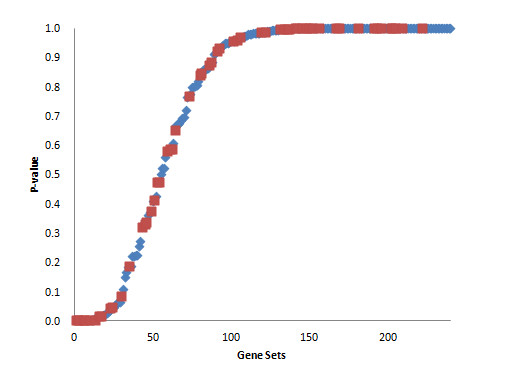
**Sorted *p*-values of gene sets of IGF-1 QTL for three comparisons**. Maximum *p*-values in the three comparisons are used. Red (blue) dots indicate the sub- (whole) pathways more significant than the whole (sub-) pathway.

The IGF-1 QTL set consisted of 3,135 genes and 80 pathways. In all four comparisons, there were only two pathways in common (Table 3). This may be due to a small phenotypic difference between B6 and NZB (Table 1). If we exclude the comparison between B6 and NZB, an additional 5 pathways were found (Table 3). Figures 2 and 3 show the maximum *p*-value distributions of IGF-1 gene sets in four and three comparisons, respectively. Most of the pathways identified were categorized as Environmental Information Processing and Organismal Systems, related to some biological processes for IGF-1 regulation. We can roughly divide them into two groups. The first group includes gene sets related to "upstream" biological processes or genes for IGF-1. Yusta et al. (2009) [19] suggested ErbB signaling to be upstream of and lead to IGF-1's activation. In liver, *FGF21 *in the MAPK signaling pathway decreases on the expression of *IGF-1 *via a reduction in concentrations of the active form of STAT5 [20]. The second group contains gene sets that are "downstream" of IGF-1. It is suggested that IGF-1 serves as an important signal in the regulation of both human and rodent GnRH gene expression [21]. These pathways appear to be linked with the MAPK signaling pathway which seems to be important for IGF-1-stimulated DNA synthesis and HGF production [22], the connection between pathways defined by the Kyoto encyclopedia of genes and genomes (KEGG) [23].

**Table 3:** The IGF-1-related pathways detected

	Map category	Direction of change in gene expression*	No. of genes	No. of genes within QTL regions
All four comparisons				
PPAR signaling pathway	Organismal Systems	U	65	11
Drug metabolism - other enzymes	Metabolism	D	36	17
Three comparisons without B6 vs NZB				
ErbB signaling pathway	Environmental Information Processing	B	71	18
GnRH signaling pathway	Organismal Systems	B	88	27
MAPK signaling pathway	Environmental Information Processing	B	234	58
Steroid hormone biosynthesis	Metabolism	D	36	12
Gastric acid secretion	Organismal Systems	D	63	16

*B, U and D represent bi-directional, up and down-regulation, respectively.

## Conclusions

In this study, we have developed a method of identifying pathways that are associated with quantitative traits using information on QTL. In the application of our approach to the data on mouse inbred strains, the metabolic pathways for HDL cholesterol trait and the IGF-1 related signaling pathways were clearly detected. Our approach provides insights into genotype-phenotype relations at the level of biological pathways which may help to elucidate the genetic architecture underlying the variation in phenotypic traits. The method presented here is also useful for identifying positional candidate genes in the QTL region through the detection of trait-associated pathways. Once trait-associated pathways are detected, differentially expressed genes that share the pathways would be promising candidates, even if the genes are outside the QTL region.

## Methods

### Current QTL/microarray approach

GSA methods can be separated into a number of discrete steps enabling systematic comparison [24]. The current approach consists of 1) the calculation of gene-level statistics, 2) the transformation of gene-level statistics, 3) the computation of a gene set statistic and 4) an assessment of significance. The first three steps are similar to two other commonly used GSA methods, GAGE [25] and PAGE [26], and therefore, were implemented using the gage R package [27]. All current analyses were conducted using two-class unpaired comparisons between different genetic strains.

1) With the current method, the simplest and most widely used fold change is calculated as a gene-level statistic by the log-ratio (base 2) of the group means of one gene.

2) In addition to an absence of transformation for the typical single direction enrichment, absolute values of per-gene test statistics were used to identify bi-directional changes, providing a more precise understanding of transcriptome regulation.

3) The most common choice for the gene set statistic is the mean of local statistics, especially when the gene-level scores are assumed to follow a normal distribution. We employed Z-scores for each gene set which were calculated from (absolute) fold change values for each gene between two experimental groups, as follows:

$$z=\left(x-\mu \right)/\sqrt{\sigma^{2}/n},$$

where *μ *and σ are the mean (absolute) fold change and the standard deviation of all genes in a given microarray, respectively, and *x *and *n *are the mean of (absolute) fold change and the number of genes in the gene (sub-)set, respectively.

4) If a pathway is associated with the trait, we expect a sub-set of pathways within the QTL regions to have altered transcription. We intersected two gene sets, each of which belongs to the pathway and QTL category so that the intersection has composite information. Statistical tests conducted use a competitive null hypothesis. Suppose there are *m *gene sets (*G_i_*) in the pathway category and these *p*-values are computed from the Z-score. For the given QTL category, the sorted *p*-values for sub-sets are

$$P\left(G_{1}\cap Q\right)\leq P\left(G_{2}\cap Q\right)\leq \cdots \leq P\left(G_{i}\cap Q\right)\leq \cdots \leq P(G_{m}\cap Q),$$

where *Q *represent genes within all QTL regions for a specific trait. We are particularly interested in the intersection sets with significant expression changes for which the changes in the individual gene sets are less significant. If we want the FDR (*α*) [28] of an entire experiment, the significance of the sub-set is calculated as

$$P\left(G_{i}\cap Q\right)<\text{min}\left\{P\left(G_{i}\right),\;\;i\alpha /m\right\},$$

where *iα/m *means the critical value in the Benjamini-Hochberg procedure. In the current approach, an additional filtering based on the comparison of significance between sub- and whole pathways is implemented, although the method is not testing any specific null hypothesis for comparison of sub- with whole pathways. This approach assumes that the sub-set of pathways within the QTL region is under cis-regulation and partial trans-regulation within multiple QTL regions.

### QTL information

We applied our methods to two sets of mouse QTL identified from a public database. We extracted 47 and 17 QTL associated with blood concentrations of HDL cholesterol and IGF-1 in mice from MGI. For HDL cholesterol, we used annotations derived from the MP term "abnormal circulating HDL cholesterol level" (MP:0000184). For IGF-1, we used annotations from the term "abnormal circulating insulin-like growth factor I level" (MP:0004700). The QTL studies, the QTL name, peak position and confidence intervals were downloaded from MGI. If QTL flanking markers were not known, we assigned an interval by peak marker ± 20 Mb. To assign base pair and cytogenetic positions to all flanking and peak markers, we downloaded the UCSC Genome Browser [29] positional tables and parsed them corresponding GeneBank accession numbers. Base pair positions of the transcript first (5') and last (3') nucleotides are compared with the QTL base pair positions on the respective chromosome. If a gene overlapped with a QTL, information for the QTL is linked with the gene data.

### Microarray data

Bennet et al. (2010) [30] compared gene expression profiles from 99 strains of inbred and recombinant inbred mice in their eQTL study. The data set came from the gene expression omnibus (GEO) [31] under accession no. GSE16780. We extracted data on C3H, B6, NZB, and SM strains including for liver tissue of 16-week-old male mice from the GSE16780 dataset. The platform for the data was the Affymetrix HT Mouse Genome 430A oligonucleotide microarray (22,716 probe sets). The data was processed using the Affymetrix GCOS algorithm utilizing the Robust Multi-array Average method (RMA) to determine the specific hybridization signal for each gene. We used expression values computed by merged triplicate arrays for the same strain with an average value. After filtering the Affymetrix control probe sets named starting with "AFFX-" which are generally used to evaluate hybridization performance and quality of the biological sample, to identify the single most appropriate probe set for each gene, the probe set with the maximum absolute expression value was uniformly assigned to the gene. In the end, we obtained 14,471 probe sets uniquely representing each gene/transcript covered by the Affymetrix HT Mouse Genome 430A array.

### Pathway information

Each gene is subsequently annotated with its associated biological pathways, obtained from KEGG, a collection of manually drawn pathway maps representing the molecular interaction and reaction networks for 1) metabolism, 2) genetic information processing 3) environmental information processing, 4) cellular processes, 5) organismal systems and 6) human diseases. We limited the gene set size between ten and five hundreds and the resulting number of gene sets was 151 with 4,115 genes on the microarray (after removal of human diseases category maps). If QTL information is about eQTL regions, other gene sets such as transcription factor-target genes are needed to consider the characteristic feature.

## List of abbreviations used

eQTL: expression quantitative trait loci; FDR: false discovery rate; GEO: gene expression omnibus; GSA: gene set analysis; GWAS: genome wide association studies; HDL: high density lipoproteins; IGA: individual gene analysis; IGF-1: insulin-like growth factor 1; KEGG: Kyoto encyclopedia of genes and genomes; MGI: Mouse Genome Informatics; MPD: Mouse Phenome Database; QTL: quantitative trait locus; RMA: Robust Multi-array Average method.

## Competing interests

The authors declare that they have no competing interests.

## Authors' contributions

HM conceived of this study, developed and implemented the statistical analysis, drafted the manuscript. YT supported the design of the experiments and the interpretation of the results. HI coordinated the study and contributed in writing the manuscript. All authors read and approved the final manuscript.
